# Ten simple rules for mentoring and being mentored while neurodiverse

**DOI:** 10.1371/journal.pcbi.1013917

**Published:** 2026-04-24

**Authors:** Adam B. Smith, Emily G. Adams, Ethan Abercrombie, Noor Bibi, Claire L. J. Bottini, Alissa J. Brown, Cybil N. Cavalieri, Alonwyn L. Clauser, Marlyse C. Duguid, Kasey D. Fowler-Finn, Jenna Hutchen, Victor Leite Jardim, Clarissa S. Rodriguez, Beck M. Swab, Steph Varghese

**Affiliations:** 1 Center for Conservation & Sustainable Development, Missouri Botanical Garden, Saint Louis, Missouri, United States of America; 2 Department of Biology, Lipscomb University, Nashville, Tennessee, United States of America; 3 Department of Biology, Saint Louis University, St. Louis, Missouri, United States of America; 4 Department of Biology, Washington University in St. Louis, Saint Louis, Missouri, United States of America; 5 Division of Biology and Biomedical Sciences, Washington University in St. Louis, Saint Louis, Missouri, United States of America; 6 Department of Biology, University of Western Ontario, Condon, Ontario, Canada; 7 Independent Researcher; 8 Department of Psychology, Shenandoah University, Winchester, Virginia, United States of America; 9 School of the Environment, Yale University, New Haven, Connecticut, United States of America; 10 Department of Biology, Saint Louis University, Saint Louis, Missouri, United States of America; 11 Department of Biology, Carleton University, Ottawa, Ontario, Canada; 12 Université de Bretagne Occidentale, Institut universitaire européen de la mer, Brest, Britagne, France; 13 Department of Biology, San Diego State University, San Diego, California, United States of America; 14 Root & Spiral, Inc, Cleveland, Ohio, United States of America; 15 Ecology, Evolution, and Behavior Department, University of Minnesota, Minneapolis, Minnesota, United States of America; 16 Department of Biological Sciences, California State University, Los Angeles, Los Angeles, California, United States of America; Dassault Systemes BIOVIA, UNITED STATES OF AMERICA

**Editor's Note**

Two authors are unable to list their professional affiliations on this article and so are listed as Independent Researchers. The authors stated that their affiliated institutions, which are known to the journal, did not provide material or other support for this work, as it was performed on their own time and with their own resources.

Additionally, two contributors, whose identities are known to the journal, requested to remain anonymous and to be acknowledged rather than listed as authors due to risks they may incur for being associated with this work; their contributions are described in the article's Acknowledgments. The journal completed checks pertaining to these institutions and individuals whose identities are not listed in the published article, and confirmed that the article and its peer review comply with PLOS policies, including on Competing Interests and Funding Disclosures.

*Let’s don’t force a square peg in a round hole; let’s create more square holes.*

*—*Gary Moore, nonPareil Institute co-founder

Neurodiversity encompasses the natural, non-pathological range of variation in human brain function, including emotion, cognition, and perception [1]. The term serves as a catch-all for many diverse identities and is often defined in juxtaposition to “neurotypicality,” which refers to a given culture’s presumed “normal” mode of behavior and brain function. While no definitive list of neurodiverse identities exists, commonly-recognized identities include autism spectrum profile, variable attention stimulus trait (VAST, also called attention-deficit/hyperactivity disorder; Box 1), sensory processing condition, obsessive compulsive profile, dyspraxia, dyslexia, dyscalculia, Tourette’s, and many more [2,3].

Society benefits from the diversity of thought, perception, and experience contributed by neurodiversity. Neurodivergent people can be detail-oriented, engaged, enthusiastic, committed, and persevering [4–8]. Yet, deviations from neurotypical behaviors often solicit negative judgments and stigma. Pressure to conform to neurotypical cultural norms (i.e., masking) creates barriers to success for neurodivergent people. Because society tends to ignore, undervalue, and pathologize neurodivergence [2,9], there is a pervasive lack of understanding of how to support neurodiverse people in education, science, and the workplace [3]. Consequently, neurodiverse people—including many of the named authors of this work, plus others who contributed but are not revealing their identities for fear of repercussions—experience excessively high rates of stress, depression, burnout, and school/workforce drop-out [5,10,11].

We outline a set of 10 simple rules for supporting neurodiversity in the workplace, with a focus on neurodiverse mentees, mentors of neurodiverse mentees, neurodiverse mentors, and allies. We also provide four simple rules for mentees of neurodiverse mentors (Box 2). As a team of neurodiverse researchers and allies, we write with personal experience while also drawing on empirical research [12].

## 1. Reorient toward a social model of neurodiversity

Neurodivergence is often cast as “disability”. For example, the American Psychiatric Association’s *Diagnostic and Statistical Manual of Mental Disorders* defines autism, variable attention, obsessive-compulsive, and other profiles in pathological terms (e.g., “attention *deficit*/hyperactivity *disorder*”) [13]. The resulting stigma often means that society overlooks the strengths of neurodivergent people and mischaracterizes adaptive behaviors. For example, a neurodivergent person may hum, ask for reminders, or seek solitude to self-soothe and reorient. From a pathological lens, these behaviors are often seen as “problems” that need to be addressed. However, from a social perspective, these typically harmless adaptations allow neurodiverse people to function in an environment stressful to them.

Endorse the social model of neurodiversity [1,14]: *Neurodiversity is the* ***natural*** *variation in human brain function and behavior. Disability is a characteristic of the environment’s “fit” to a neurodivergent person’s needs.* The person is not the problem; disability arises from context.

## 2. Recognize that neurodiversity is *diverse*!

**Fig 1:**
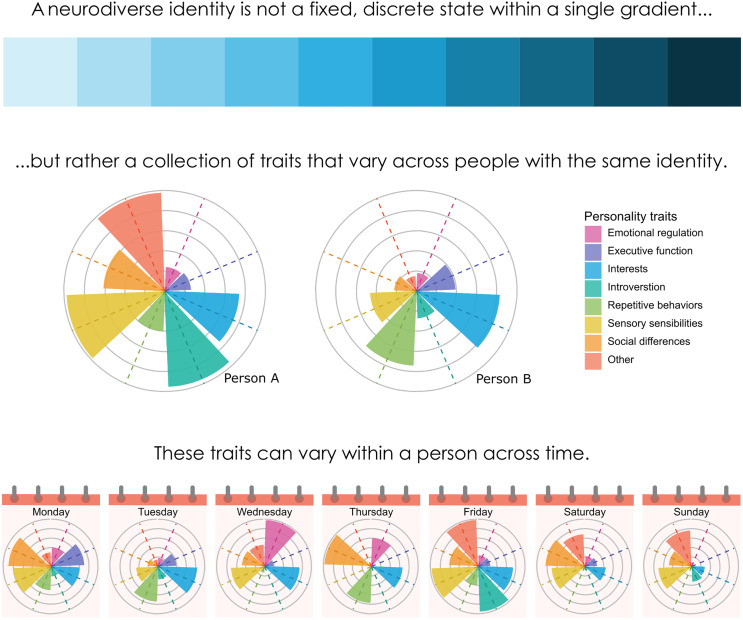
Neurodiversity is not a linear gradient from “typical” to “diverse” on which a person can be placed. Inspired by work by Instagram user autism_sketches.

A common refrain in the neurodiverse community asserts, “If you’ve met one person with *<some neurodiverse identity>*, you’ve met just one person.” Indeed, there are scores of neurodiverse profiles, and within profiles, people can diverge widely. For example, obsessive compulsive profile can be further divided into up to 10 variants, with differing emphases on precision and cleanliness. Neurodivergent people can also feel more or less neurodivergent on different days and across different environmental and social contexts, similarly to the way a neurotypical person feels changes with time (Fig 1). Approach every person and situation open-mindedly.

## 3. Recognize that neurodiversity is often hidden

Neurodivergence in people around you will often be hard to identify. Many neurodivergent people choose not to self-disclose for fear of being stigmatized [11,15]. Pressure to conform to neurotypical standards encourages a person to “mask” their neurodivergent traits. People may also not be aware of their own neurodivergence [16]. Indeed, across our author team, several of us were only diagnosed as adults.

## 4. Address common barriers and provide accommodations to everyone

If neurodiversity is often hidden, how can we accommodate it? The answer is to extend accommodations to all. Inform yourself about barriers faced by neurodivergent people and work to remove those barriers for everyone—openly neurodivergent or not. Providing accommodations to everyone helps everyone, neurodiverse or neurotypical. For example, establishing a policy to “take extended conversations outside a shared office” can reduce distracting side chatter that benefits everyone concerned. Similarly, training individuals on time management or other executive functioning tasks, and conveying the same information in multiple modes (verbal, written, visual, etc.) assists neurotypicals and neurodivergents alike [17–20].

## 5. Build on pre-existing strengths

The pathological model of neurodiversity focuses on disadvantage and dissuades seeing neurodivergent characteristics as assets [21]. For example, in comparison to neurotypicals, people with obsessive-compulsive profiles can be inherently focused thinkers [22], dyslexics can display better skill with visual-spatial tasks [23,24], and dyspraxics can be more detail-focused and innovative [25]. Mentors should provide opportunities for leveraging neurodiverse strengths. These opportunities should allow for meaningful growth—i.e., the foundational skills of research: designing and executing workflows and experiments; qualitative/quantitative analysis; mentoring; and professional writing, reading, and presenting. For example, a detail-oriented person could be put in charge of performing complex lab procedures that contribute to their own work, and especially creative people can be very helpful troubleshooters when procedures fail.

This said, avoid seeing neurodivergent people only for their strengths. Doing so is a commodification of a person that usurps actual inclusion [26,27]. Value neurodivergent people because they are people.

## 6. Define progress on individual terms

Tools and metrics of success that work for one person may not work for another. Specifically, many neurodivergents (and neurotypicals) benefit from coaching on strategies for executive function (planning, organizing, initiating, and completing tasks). What one person can do easily may take another person extreme, concerted effort. Indeed, many neurodiverse identities can be described in terms of “interest-based” tendencies. For example, a person may find fascination in complex statistical manipulations and have no challenges meeting milestones when implementing workflows around these, but struggle with similarly complex lab work. These tendencies cut across the neurodiverse/neurotypical divide, but can be more pronounced in the former.

Defining progress on individual terms does not mean exercising favoritism or lower standards. Indeed, neurodiverse people often prefer what might be perceived as higher standards (i.e., “hard” deadlines—versus time-flexible ones) [28].

## 7. Embrace your own neurodivergent identity

For neurodiversity to become normalized, people in positions of power need to recognize and value it, which in turn encourages neurodivergent mentors and mentees to be more open about their own identities and struggles. Consider how you can embrace and embolden your own identity. We do not mean that people should always uncloset themselves. Indeed, there are real social costs to disclosure (though also benefits, such as enhanced support). Rather, self-disclosure can vary in degree and kind, and range from confiding in trusted colleagues, establishing a neurodiverse-friendly lab, hosting seminars on how to empower neurodiversity in the classroom, inviting guest speakers to educate on neurodiversity, to openly talking about one’s own struggles and path to self-knowing.

The notion of “niche-building” is useful, wherein individuals shape their environment to match their genotypes [29]. Neurodiverse people are not simply “receptors” of social and environmental conditions—they transform them. Embracing you identity is a form of niche building. There is power in this.

## 8. Create a culture supportive of neurodiversity

**Fig 2:**
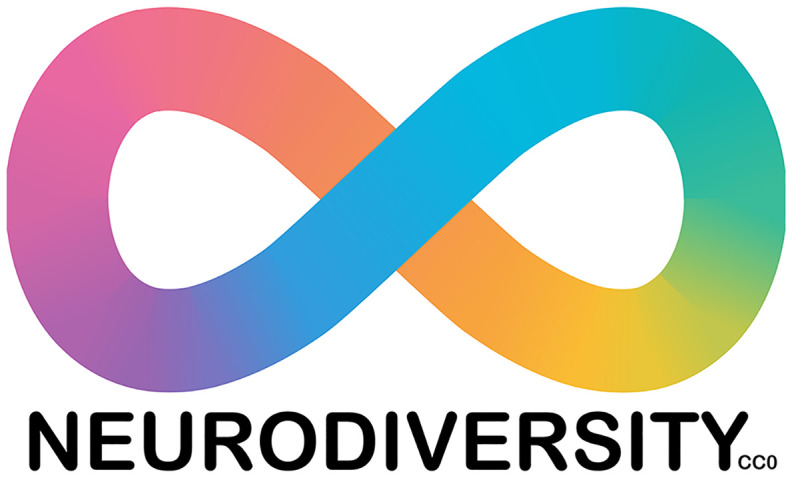
Advertise the neurodiverse-friendliness of your workplace by displaying a sign to that effect, like the one in this figure, which is licensed under the Creative Commons CC0 1.0 Universal Public Domain Dedication (free to use, no attribution necessary, no restrictions on adaptation or reuse). The one-sided Möbius strip was created by Wikimedia contributor MissLunaRose12, an autistic artist.

Mentors, advertise that you run a neurodiverse-friendly workplace, for example, by putting a statement on your team’s website (Fig 2) where you describe your approach and motivation to be neurodiverse-friendly in a mentoring statement (you do have a mentoring statement, don’t you?). Have a conversation about neurodiversity with *everyone*, not just mentees who have self-identified. Within your team, model the behavior you want to see. For example, a mentee may come to you to complain about another lab member’s actions (e.g., humming to themselves or blunt communication style). The pathological model approach would seek to “solve this problem”: commanding the subject of complaint to “stop it” or “fix it.” A social model approach would start with curiosity: *Why* is this behavior happening? Perhaps, for example, the humming is stimming (behavior that reduces stress). Is there an accommodation that could help? Seek solutions that are equitable (not necessarily “equal”).

Work with all mentees to make sure that they have the support they need to be successful regardless of identity (offer accommodations to everyone—rule #4). Interpersonal struggles are an excellent teaching moment for mentees, as they are not only training to do science, but also to work as part of a team. Focus on motivations, not identity. For example, the complaint “You’re too obsessive and try to control everything” critiques the individual, whereas the observation that “I can see that you are concerned the analysis won’t succeed, so seem to be stepping in a lot to direct the work” acknowledges the motivation behind the behaviors.

## 9. Seek outside assistance

Resources will vary by country and institution, so we can only provide general advice, but getting external support is essential to living as a neurodiverse person and maintaining a neurodiverse-friendly workplace. External support can include therapy, coaching, support groups, mentoring, and other informal structure. Universities and organizations can get institutional memberships to groups like the National Center for Faculty Development and Diversity, which supports scholars and provide trainings that are helpful to all identities. At the individual level, many online and in-person coaching programs specifically target neuro-divergent scholars. Podcasts such as *NeuroDiving* offer expert advice and support.

Nearly every neurodiverse identity has a formal society and informal affinity groups. For example, the Center for the Integration of Research, Teaching, and Learning (https://cirtl.net) holds workshops and has resources for inclusive teaching and professional development within the academic workplace. Likewise, the Society for Neurodiversity (https://s4nd.org) advocates for civil rights of neurodivergent people. Identity-specific groups are also numerous and include the Autistic Self Advocacy Network (https://autisticadvocacy.org), the Attention Deficit Disorder Association (https://add.org), and the International Obsessive Compulsive Disorder Foundation (https://iocdf.org), amongst many others. Some universities also have campus-specific support groups.

## 10. Change the institution, then the world

Institutions must actively support neurodiversity for its potential—and the humanity behind it—to be realized. Administrators, start by building specific trainings and providing ongoing, institutional support for mentors. While education and awareness will always be essential, alone, they are not enough. Key systems and processes need to be redesigned. Meeting legal requirements is the bare minimum—aim high. Institutions need to provide support for neurodiverse mentees *and* mentors. A one-stop support system works better than piecemeal procedures. Advertise these supports; don’t let them go hidden to those who need them most.

## Conclusion

In some countries, societal headwinds are blowing against efforts to acknowledge and increase support for diversity [30]. This is exactly the time champions are needed. Even a sailboat can tack against the wind.

There are many routes to success, including those that deviate from neurotypical archetypes. Identifying and building on these strengths is a worthwhile challenge. Although the material gains of adopting a pro-neurodiverse approach are many, in the end, neurodiverse people should be supported first and foremost because they are individuals with inherent worth [31].

**Box 1. How to talk *about* neurodiversity: The no-rule rule**

Within the neurodiverse community, there is a range of opinions on how to talk about neurodiversity. For example, some prefer person-first nomenclature, as in “a person with autism” versus diagnosis-first, such as “an autistic person.” Likewise, particular identities can have alternative names. For example, “variable attention stimulus trait” (VAST) has been proposed to take the place of “attention-deficit/hyperactivity disorder (ADHD)”, because the latter is burdened with connotations of pathology. Even within our authorship team, we disagree over which terms are better!

How should you talk about neurodiversity? We recommend a “no-rule” rule: **if you are speaking with a person with a particular neurodiverse identity, ask them what phrasing they prefer**. Let them state the language that works for them, and honor it, just like you would use their preferred name. When speaking with multiple neurodiverse people or to groups, acknowledging the options can be a good place to start.

What if you mess up? It’s OK, we all do. Apologize and move on. Making a deal of it can impose a social obligation to console you and shift the focus. Have grace with others, and with yourself.

**Box 2. Four simple rules for mentees of neurodiverse mentors**

For mentees working with neurodiverse mentors, many of the same principles outlined in the main text still apply, but we place a particular emphasis on external support. Other lab members or peers familiar with your mentor can provide a circle of support.

1. **Resist the impulse to pathologize.** As a mentee, your role is not to diagnose your mentor. Focus on your mentor’s strengths and use them as a foundation for your relationship. Have grace: no mentor, neurodiverse or not, will perfectly align with your needs. Actively develop complementary supports. This could involve recruiting additional mentors who can offer further support.
2. **Work towards a shared goal**. It may be helpful to support your mentor through certain dynamics, but always approach this with sensitivity and respect. Frame your suggestions as tools for mutual problem-solving, not correction. Meeting halfway with empathy and clarity builds trust.
3. **Feedback**—**especially direct, respectful feedback**—**is essential.** Rather than assuming your mentor “should know” how they are affecting others, it can be helpful to view feedback as a bridge toward mutual understanding.
4. **Don’t lose sight of your own needs**. Advocate for yourself. You have boundaries that are important to maintain, regardless of the reason for specific behaviors of your mentor. You are not responsible for their emotions, nor must you accommodate every difference. Clearly articulating your needs (and encouraging your mentor to do the same) can help mitigate potential challenges.
